# Ketamine Use allows Noninvasive Ventilation in Distressed Patients with Acute Decompensated Heart Failure

**DOI:** 10.5005/jp-journals-10071-23153

**Published:** 2019-04

**Authors:** Ankur Verma, Abhishek Snehy, Amit Vishen, Wasil Rasool Sheikh, Meghna Haldar, Sanjay Jaiswal

**Affiliations:** 1–6 Department of Emergency Medicine, Max Super Specialty Hospital, Patparganj, New Delhi, India

**Keywords:** Dissociation, Heart failure, Ketamine, NIPPV

## Abstract

Acute decompensated heart failure (ADHF) is responsible for a heavy clinical load on busy emergency departments (EDs) across the globe and especially in India. ADHF patients may present with severe respiratory distress, dyspnea, hypoxia, and high and low blood pressures. Managing the airway of such patients can at times be challenging. Nasal cannulae, face mask, and noninvasive positive pressure ventilation (NIPPV) are the cornerstones of providing oxygenation and ventilation to such patients while some extreme cases may require endotracheal intubation and mechanical ventilation.

An elderly female in severe respiratory distress and altered sensorium presented to our ED and had to be administered ketamine to facilitate proper NIPPV and avoid mechanical ventilation. She was weaned off the NIPPV in the ED itself over the next four hours. There are some case reports of using ketamine for NIPPV in asthma exacerbations, but none for the use in ADHF. Avoiding invasive mechanical ventilation via endotracheal intubation should be a constant goal and the last resort.

**How to cite this article:** Verma A, Snehy A, *et al*. Ketamine Use Allows Noninvasive Ventilation in Distressed Patients with Acute Decompensated Heart Failure. Indian J Crit Care Med 2019;23(4): 191–192.

## INTRODUCTION

Any acute or insidious progression of heart failure (HF) requiring urgent medical attention is what defines ADHF.^1^ It is responsible for a heavy clinical load and increases the burden on healthcare and EDs across the globe. Even after the availability of evidence-based medical therapies using nitroglycerine, loop diuretics, levosimendan, etc., the in-hospital mortality across the United States and Europe is 4 – 7%.^2–4^ The acute failure registry (AFAR) study conducted in India showed a 38% mortality of ADHF patients admitted.^1^ Dyspnea is usually the most common symptom with which patients of ADHF present to the ED. Patients can present in altered mental status (AMS) due to hypoxia, which can make standard therapies like noninvasive ventilation and their outcomes difficult to achieve. Patients with ADHF presenting in respiratory distress will require stabilization of their airway and breathing. Oxygen supplementation, especially through NIPPV, has been shown to reduce the need for intubation.^5^ We describe a case where ketamine was used to allow compliance with NIPPV in a severely distressed patient of ADHF.

## CASE HISTORY

A 71-year-old female presented to our ED in severe respiratory distress and AMS. She was a known diabetic, hypertensive, post permanent pacemaker implantation, and was on diuretics and angiotensin-converting enzyme (ACE) inhibitors for dilated cardiomyopathy with a baseline ejection fraction (EF) of 25%. On arrival she was tachypneic with respiratory rate of 40 breaths/minute, heart rate of 120 beats/minute, blood pressure 200/110 mm Hg, and oxygen saturation as indicated by pulse oximeter was 75% on room air. Her temperature was 98°F and random blood sugar was 156 mg/dL. Systemic examination revealed reduced air entry in both lungs and coarse crepitations bilaterally. Oxygen supplementation was started at 8 L/minute via reservoir mask. Intravenous furosemide 60 mg and nitroglycerine infusion (20 µg/minute) were given. She did not respond to oxygen supplementation and decision to start her on bilevel positive airway pressure (BiPAP) ventilation was made. Consent for intubation was also taken from the family. Attempts to start BiPAP failed as the patient would push away the mask and was increasingly agitated. Intravenous bolus dose of 30 mg ketamine, which lightly sedated the patient, was given and BiPAP was started. After about 30 minutes the patient awoke and was given another bolus of 15 mg of ketamine while the patient was still on BiPAP. One hour later, patient was conscious and oriented and showed good tolerance to BiPAP. Her respiratory rate settled to 16 breaths/minute with saturation of 95% on BiPAP, heart rate of 78 beats/minute, and blood pressure of 130/70 mm Hg. Four hours after arrival to the ED she was weaned off the BiPAP and nitroglycerine and maintained oxygen saturation of 100% on 2 L/minute via nasal cannula, respiration of 16 breaths/minute, heart rate 68 beats/minute, and blood pressure of 140/80 mm Hg. The initial electrocardiogram (ECG) had shown a paced rhythm with no acute changes. Two-dimensional echocardiography done in the ED revealed global hypokinesia with EF of 25%. Blood gas at the time of arrival revealed severe type 1 respiratory failure. Laboratory tests were significant for N-terminal pro-brain natriuretic peptide (NT-ProBNP) of 13,900 pg/mL. She was shifted to the cardiac unit for further management. The patient was managed conservatively and was discharged in a stable condition after 3 days on medications and follow-up advice.

## DISCUSSION

Ketamine and NIPPV have been used in asthma exacerbations as a temporizing measure to avoid mechanical ventilation.^6^ Although not many studies have been done, there are reports^6,7^ where the use of ketamine has facilitated the use of NIPPV and the need for endotracheal intubation has been avoided in such patients. It has also been reported that patients presenting to the ED in respiratory distress when induced with ketamine for intubation have responded to oxygenation with NIPPV and did not require endotracheal intubation.^7^ There have been no case reports or studies of having used ketamine in HF patients to allow compliance with NIPPV. Although there is a level B evidence, as described by the American Heart Association Scientific statement^8^ that ketamine can precipitate HF due to its negative inotropic effects, the evidence remains inconclusive. There have been theoretical concerns that use of NIPPV in patients with AMS increases the risk of aspiration.^9^ however, ketamine-induced sedation leads to the retention of airway reflexes and spontaneous breathing.^10^ Agitated and restless patients presenting with severe respiratory distress can pose a challenge to manage. If a patient does not comply with NIPPV, unwanted endotracheal intubation might be required in the ED.

We believe that ketamine induced dissociative state in the patient with ADHF facilitated NIPPV management in an otherwise uncooperative patient and allowed NIPPV to take effect. This led to the avoidance of an impending intubation. The patient had an uneventful stay in the hospital which could be attributed to the acute management in the ED. As emergency physicians, it should be our goal to avoid intubating patients and putting them on mechanical ventilation when other options may be attempted. Large series and trials would be required to establish the use of ketamine induced sedation in agitated, uncooperative patients of HF to allow compliance with NIPPV. Till then we suggest it to be used with caution in this subset of patients.
